# Rumen fermentation and microbial diversity of sheep fed a high-concentrate diet supplemented with hydroethanolic extract of walnut green husks

**DOI:** 10.5713/ab.23.0213

**Published:** 2023-11-02

**Authors:** Huan Wei, Jiancheng Liu, Mengjian Liu, Huiling Zhang, Yong Chen

**Affiliations:** 1 Laboratory of Nutrition for Meat & Dairy Herbivore, College of Animal Science, Xinjiang Agricultural University, Urumqi 830052, China

**Keywords:** Hydroethanolic Extract, Microbial Community, Rumen Fermentation, Subacute Rumen Acidosis, Volatile Fatty Acids, Walnut Green Husks

## Abstract

**Objective:**

This study aimed to assess the impact of a hydroethanolic extract of walnut green husks (WGH) on rumen fermentation and the diversity of bacteria, methanogenic archaea, and fungi in sheep fed a high-concentrate diet.

**Methods:**

Five healthy small-tailed Han ewes with permanent rumen fistula were selected and housed in individual pens. This study adopted a self-controlled and crossover design with a control period and an experimental period. During the control period, the animals were fed a basal diet (with a ratio of concentrate to roughage of 65:35), while during the treatment period, the animals were fed the basal diet supplemented with 0.5% hydroethanolic extract of WGH. Fermentation parameters, digestive enzyme activities, and microbial diversity in rumen fluid were analyzed.

**Results:**

Supplementation of hydroethanolic extract of WGH had no significant effect on feed intake, concentrations of total volatile fatty acids, isovalerate, ammonia nitrogen, and microbial protein (p>0.05). However, the ruminal pH, concentrations of acetate, butyrate and isobutyrate, the ratio of acetate to propionate, protozoa count, and the activities of filter paper cellulase and cellobiase were significantly increased (p<0.05), while concentrations of propionate and valerate were significantly decreased (p<0.05). Moreover, 16S rRNA gene sequencing revealed that the relative abundance of rumen bacteria *Christensenellaceae* R7 group, *Saccharofermentans*, and *Ruminococcaceae* NK4A214 group were significantly increased, while *Ruminococcus gauvreauii* group, *Prevotella* 7 were significantly decreased (p<0.05). The relative abundance of the fungus *Pseudomonas* significantly increased, while *Basidiomycota*, *Fusarium*, and *Alternaria* significantly decreased (p<0.05). However, there was no significant change in the community structure of methanogenic archaea.

**Conclusion:**

Supplementation of hydroethanolic extract of WGH to a high-concentrate diet improved the ruminal fermentation, altered the structure of ruminal bacterial and fungal communities, and exhibited beneficial effects in alleviating subacute rumen acidosis of sheep.

## INTRODUCTION

Cattle and sheep producers often feed their animals high concentrate diets to achieve higher economic returns. Non-structural carbohydrates such as starch are rapidly hydrolyzed by rumen microorganisms to produce lactate and volatile fatty acids (VFAs). When the rumen absorption rate is lower than the production rate, organic acids accumulate and the pH of the rumen fluid decreases. Subacute rumen acidosis (SARA) occurs when rumen pH is depressed below 5.6 and lasts for more than 3 hours per day. Subacute rumen acidosis results in an alteration of rumen microflora, inflammation of tissues and organs, and even causes host diarrhea and dehydration [1]. Previous research has reported that some plant extracts can regulate ruminal fermentation, increase rumen pH, and prevent SARA in ruminants. Plant-derived feed additives such as extracts of cinnamon, grape seed, orange, pomegranate peel have beneficial effects on reducing SARA induced by high concentrate feed [2]. Plant extracts are rich in active ingredients such as saponins, essential oils, and phenolic compounds (such as tannins and flavonoids), which have multiple effects such as anticancer, anti-inflammatory, antioxidant, and antibacterial. Some plant extracts show beneficial effects in improving rumen fermentation and preventing rumen acidosis and are potential antibiotic alternatives.

Walnut (*Juglans regia* L.) green husk (WGH) contains many active compounds such as polyphenols, flavonoids, naphthoquinones, and their derivatives [3], and is an economical source of antioxidants and antimicrobials [4]. The ethyl acetate extract of WGH can reduce the production of lactate and VFAs by decreasing the activities of key enzymes of lactate synthesis and glucose metabolism, thereby increasing the pH of rumen fluid, improving rumen fermentation, and alleviating SARA induced by high concentrate feeding of sheep [5]. It is not clear whether hydroethanolic extract of WGH also has the beneficial effect on rumen fermentation in sheep, and no effect on rumen microbial diversity has been reported. Therefore, in this study, hydroethanolic extract of WGH was added to the diet to investigate its effects on rumen fermentation and microbial diversity, to provide a theoretical basis for the practical application of WGH extract as a feed additive in the prevention of SARA in sheep.

## MATERIALS AND METHODS

### Preparation of hydroethanolic extract of walnut green husk

The fresh walnut “Wen185” from Aksu Prefecture of Xinjiang in China was collected, and the WGH was peeled, air-dried, and crushed through an 80-mesh sieve using a grinder (DF-20; Wenling Dade Traditional Chinese Medicine Machinery Co., Ltd, Wenling, China). Ethanol-water at 25:75 (v/v) was used as the extraction solvent, and the ratio of husk powder to extraction solvent was 1:10 (w/v). The WGH was extracted in a shaking incubator (ZWY-100D; Zhicheng Instrument Manufacturing Co., Ltd., Shanghai, China) at 37°C for 8 h and then filtered through a bag filter (Φ = 5 μm) to remove the residue. The filtrates were evaporated under vacuum in a rotary evaporator (RE-5210A; Shanghai Ya-Rong Biochemical Instruments Plant, Shanghai, China) in a water bath (40°C) at a rotation speed of 170 rpm, and the concentrates were dried at 50°C to constant weight and stored at −20°C.

### Animals, diets and feeding management

All experimental procedures involving animals in this study were approved by the Animal Care Committee of Xinjiang Agricultural University (No. 2022042). Five healthy non-pregnant small-tailed Han ewes with an average body weight of 40 kg and permanent rumen fistula were selected and housed in individual pens. The basal diet with a concentrate to roughage ratio of 65:35 was thoroughly mixed and pelleted to 0.5 cm in diameter and 1.0 cm in length. The composition and nutrient levels of the basal diet are shown in Table 1.

This experiment adopted a self-controlled and crossover design and was divided into a control period and an experimental period. During the control period, the animals were fed a basal diet, and during the treatment period, the animals were fed the basal diet supplemented with 0.5% hydroethanolic extract of WGH. Each period consisted of 12 d of diet adaptation, followed by 3 d of rumen content collection. During the experiment, sheep were fed daily at 09:00 h and 19:00 h with free access to feed and water.

### Sampling

During the experiment, 500 g of basal diet was collected at each period and stored at room temperature for subsequent analysis of nutrient contents. During the rumen fluid sampling period, 100 g of rumen contents were collected via the rumen fistula before feeding (0 h), and at 1.5, 3, 6 and 10 h after morning feeding, respectively. Samples were strained through 4 layers of cheesecloth and pH was measured immediately. Rumen fluid samples at each sampling time from 3 consecutive days were mixed with the same volume and stored at −20°C for subsequent analysis of VFAs, microbial crude protein (MCP), protozoal count, activity of digestive enzymes, and high-throughput sequencing of 16S rDNA and internal transcribed spacer (ITS1).

### Assays of samples

*Active ingredients of hydroethanolic extract of WGH*: The total phenols content in the hydroethanolic extract of WGH was determined by the Fohn-Ciocalteu colorimetric method [6]. Flavonoids content was determined by aluminum nitrate color development method [7]. 2,2-Diphenyl-1-picrylhydrazyl (DPPH) scavenging activity was assayed according to the method of More et al [8]. The polysaccharide content was measured by the phenol-sulfate method [9]. Active ingredients and free radical scavenging rate of hydroethanolic extract of WGH are given in Table 2.

*Nutrients in the diet*: Diet samples were analyzed according to AOAC methods [10]: dry matter (DM, Code 930.15), organic matter (OM, Code 942.05), crude protein (CP, Code 990.03), calcium (Ca, Code 920.39), and phosphorus (P, Code 946.06). Gross energy was determined using an automated adiabatic oxygen bomb calorimeter. Dietary neutral detergent fiber and acid detergent fiber were determined according to the procedures of van Soest et al [11].

*Fermentation parameters and protozoa count*: The pH of ruminal fluid was measured using a pH meter (model FE20; METTLER TOLEDO, Shanghai, China). Ammonia nitrogen (NH_3_-N) concentration was measured by the colorimetric method as described by Chaney and Marbach [12]. Briefly, 1.0 mL of rumen fluid was centrifuged at 15,000×g for 10 min at 4°C, 0.2 mL of supernatant was collected, and 0.2 mol/L HCl was added to 1.0 mL and mixed by vortexing. A portion of 0.1 mL acidified rumen fluid supernatant was taken, and 0.5 mL of distilled water, 2 mL of sodium nitroprusside - sodium salicylate solution, 2 mL of sodium hypochlorite-NaOH solution were added, mixed by vortexing, and allowed to stand at room temperature for 10 min. The optical density at 700 nm was the read on a microplate reader (Infinite M200; Tecan, Männedorf, Switzerland). The NH_3_-N concentration was calculated using NH_4_Cl as the standard. The VFAs in rumen fluid were determined by a gas chromatography (GC-2010; Shimadzu, Kyoto, Japan) equipped with a capillary chromatography column (Stabilwax-DA, 30 m×0.25 mm ID, 0.25 μm film thickness; RESTEK, Bellefonte, PA, USA) as previously described by Wei et al [13], using 4-methylvaleric acid as an internal standard. In short, 1.0 mL of rumen fluid was centrifuged at 15,000×g for 5 min at 4°C. Then, 0.5 mL of supernatant was collected and 0.5 mL of 10% (w/v) trichloroacetic acid was added, mixed by vortexing, left at room temperature for 20 min and then centrifuged at 20,000×g for 15 min at 4°C to remove protein. Subsequently, an aliquot of 0.8 mL supernatant was taken and 0.1 mL of 40 mmol/L 4-methylvaleric acid was added, mixed by vortexing, and then measured on the GC. The GC running temperature protocol was as follows: the injector was set at 230°C, the column oven was initially set at 55°C, then heated to 200°C at a rate of 13°C/min and held for 0.5 min, and the thermal conductivity detector was set at 240°C.

Microbial crude protein was assayed according to Makkar et al [14]. In brief, 5.0 mL of rumen fluid was taken and shaken on a magnetic stirrer for 45 s to release the microbes adsorbed on the feed particles. The mixture was then centrifuged at 408×g for 5 min to remove protozoa and feed particles. One millilitre of the supernatant was transferred to a new tube and centrifuged at 25,000×g at 4°C for 20 min to pellet the microbial cells. The resulting pellets were washed twice with sterile saline and resuspended in 3.0 mL of 0.25 mol/L NaOH solution. The suspension was then bathed in boiling water for 10 min, cooled, and subsequently centrifuged at 25,000×g for 20 min at 4°C. An aliquot of 0.1 mL of the supernatant was collected, and the protein was determined by the method of Bradford et al. with bovine serum albumin as the standard [15]. The optical density at 595 nm was determined using the Infinite M200 microplate reader.

The number of protozoa was counted according to the method of Dehority [16]. A volume of 5.0 mL of rumen fluid was taken, and 10.0 mL of methyl-green formalin saline staining solution (100 mL 35% formalin, 3.0 g sodium chloride, 0.6 g methyl-green, 900 mL distilled water) was added, gently shaken, and stained for 10 min. An aliquot of 0.1 mL of the stained sample was aspirated and counted under a microscope using a modified red blood cell counting plate (100× magnification).

*Activity of digestive enzymes in rumen fluid*: The activities of carboxymethylcellulase (CMCase), filter paper cellulase (FPase), cellobiase, xylanase, β-glucanase and amylase in rumen fluid were determined according to the method of Yu et al [17]. Briefly, 2.0 mL of rumen fluid was collected and centrifuged at 20,000×g at 4°C for 10 min. An aliquot of 0.1 mL of the supernatant was mixed with 0.5 mL of pre-warmed substrate solution (39°C) by vortexing, and then kept in a water bath at 39°C for 5 min. The reaction was stopped by adding 1.0 mL of 3.5-dinitrosalicylic acid (DNS) solution and then bathed in boiling water for 5 min. After cooling under running water, 3.4 mL of ddH_2_O was added and centrifuged at 4,000×g for 10 min at room temperature. The optical density of the supernatant was determined at 540 nm using the Infinite M200 microplate reader. The substrates for the activities of CMCase, FPase, cellobiase, xylanase, β-glucanase, amylase were 1% sodium carboxymethylcellulose (CMC-Na) (w/v, CAS No.:9004-32-4; Sigma-Aldrich, Munich, Germany), 1% ball-milled filter paper (w/v, Whatman No.1; GE Healthcare, Piscataway, NJ, USA), 1% salicin (w/v, CAS No.:138-52-3; Sigma-Aldrich, Germany), 1% xylan (w/v; Megazyme, Bray, Ireland), 1% barley β-glucan (w/v, CAS No.:9041-22-9; Sigma-Aldrich, Germany) and 1% soluble starch (EC No.: 232-679-6; Sigma-Aldrich, Germany), respectively. The substrate solutions were prepared with 0.2 mol/L phosphate buffer (pH 6.0). The standard substances for the enzyme activity assays were glucose for CMCase, FPase, cellobiase and β-glucanase, xylose for xylanase, and maltose for amylase, respectively. One unit (U) of enzyme activity is defined as the amount of enzyme in 1.0 mL of rumen fluid required to liberate 1 μmol of glucose, xylose, or maltose under standard assay conditions.

*High-throughput sequencing and microbial diversity analysis*: Genomic DNA of ruminal microorganisms was extracted by cetyltrimethylammonium bromide (CTAB) solution method [18] with some modifications as described in the previous study [19]. The qualified genomic DNA was used as a template to amplify the V3–V4 region of the bacterial 16S rRNA gene, the V8 region of the 16S rRNA gene of methanogenic archaea, and the ITS1 region of fungi with the barcoded primer pair. The polymerase chain reaction (PCR) primer sequences are listed in Table 3. PCR amplification was performed using Phusion High-Fidelity PCR Master Mix with GC Buffer (New England BioLabs, Ipswich, MA, USA). The PCR amplicons were purified using the GeneJET Gel Extraction Kit (Thermo Scientific, Waltham, MA, USA), sequencing libraries were constructed using the Ion Plus Fragment Library Kit (Thermofisher, Waltham, MA, USA), and high-throughput sequencing was performed on the Ion S5 XL platform (Thermofisher, USA) according to the standard protocols provided by Novogene Bioinformatics Technology (Biejing, China).

Raw sequence reads were quality controlled using the UCHIME algorithm ( http://www.drive5.com/usearch/manual/uchime_algo.html ) to generate clean reads. All clean reads were clustered using the Uparse software (version 7.0.1, http://drive5.com/uparse/). Sequences with 97% similarity were assigned to the same operational taxonomic unit (OTU). One representative sequence from each OTU was selected for taxonomic annotation. Annotation was performed for bacteria and methanogenic archaea using the Mothur algorithm with the 16S rRNA of the Silva 132 database (http://www.arb-silva.de/) [23], and for fungi using the Blast method (http://qiime.org/scripts/assign_taxonomy.html) in Qiime software (version 1.9.1) [24] with the UNITE database (https://unite.ut.ee/) [25] accessed on March 2023. After data normalization, microbial abundance indices including the number of OTUs, Chao1 and the abundance-based coverage estimator (ACE), and diversity indices including the Shannon, Simpson, and phylogenetic diversity (PD) whole tree were calculated using Qiime software. Beta diversity was evaluated according to weighted UniFrac distances and presented using principal coordinate analysis (PCoA) (https://cran.r-project.org/web/packages/GUniFrac/index.html). Venn diagrams of groups based on OTUs and PCoA plots were generated using R software.

### Statistical analysis

Significance analyses of fermentation parameters, enzyme activities, and microbial diversity indices were performed using paired-samples t-test with SPSS 26.0 software (IBM, Armonk, NY, USA), and p<0.05 was considered a significant difference. Analysis of similarity (Anosim) was used to evaluate the significance of differences in microbial community structure between groups using Bray-Curtis rank test with R software. The LEfSe (LDA effect size) software was used to identify the microbial biomarkers with significant differences between the groups [26]. In brief, the Kruskal-Wallis test was first used to identify the statistically significant taxa based on p<0.05, and the biomarkers were then screened from the statistically significant taxa based on the LDA score. The LDA score was set by default.

## RESULTS

### Voluntary intake and ruminal fermentation

The effects of hydroethanolic extract of WGH on voluntary intake and ruminal fermentation are presented in Table 4. The average daily feed intake was 1.92 kg in the control period and 2.04 kg in the treatment period, with no significant difference between groups (p>0.05). The addition of hydroethanolic extract of WGH had no significant effect on feed intake, total VFA, isovalerate, NH_3_-N and MCP compared to the control period (p>0.05). Ruminal pH, acetate, butyrate, isobutyrate, acetate/propionate, and protozoal count in rumen fluid were significantly higher than in the control period (p<0.05); propionate and valerate were significantly lower than in the control period (p<0.05).

### Activity of digestive enzymes

The effects of hydroethanolic extract of WGH on digestive enzyme activity in rumen fluid are presented in Table 5. The inclusion of hydroethanolic extract of WGH resulted in a significant increase in the activity of FPase and cellobiase (p<0.05), and xylanase activity tended to increase (p = 0.072) compared to the control period. However, no significant impact on the activity of CMCase, β-glucanase, and amylase in the rumen fluid was detected (p>0.05).

### Ruminal microbial diversity

After high-throughput sequencing and quality control, each sample yielded an average of 71,656 clean reads for bacterial 16S rDNA with an average sequence length of 418 bp, 81,600 clean reads for methanogenic archaeal 16S rDNA with an average sequence length of 280 bp, and 80,532 clean reads for fungal ITS with an average sequence length of 215 bp. The effects of the hydroethanolic extract of WGH on the diversity indices of the ruminal microbiome are presented in Table 6. The addition of hydroethanolic extract of WGH had no significant effect on the bacterial diversity indices (p>0.05). However, the observed species, Shannon, Chao1, and ACE indices of methanogenic archaea decreased significantly (p< 0.05), and the Simpson and PD whole tree indices showed a decreasing trend (p = 0.083 and p = 0.051). The observed species, Chao1, ACE, and PD whole tree indices of fungi decreased significantly (p<0.05), while there was no significant change in Shannon and Simpson (p>0.05).

**Figure 1:**
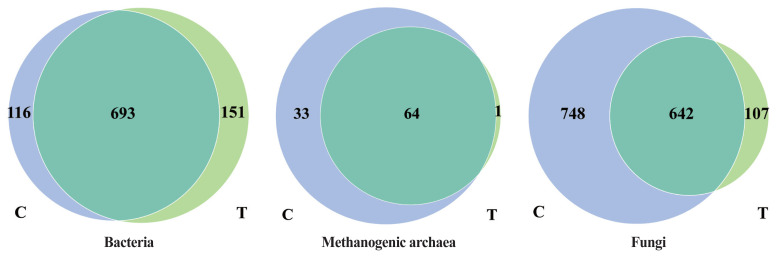
Effects of hydroethanolic extract of WGH on OTUs of rumen microorganisms in sheep. C means the control period, the animals were fed a basal diet; T means the treatment period, the animals were fed the basal diet supplemented with 0.5% hydroethanolic extract of WGH. Numbers in the gray-blue area indicate the unique OTUs found only in the control period, numbers in the green area indicate the unique OTUs found only in the treatment period, and numbers in the intersection area indicate the OTUs shared by both periods. WGH, walnut green husk; OTUs, operational taxonomic units.

The effects of the hydroethanolic extract of WGH on the OTUs of rumen microorganisms are shown in Figure 1. For bacteria, a total of 693 OTUs were shared by both periods, while 116 and 151 unique OTUs were found in the control and treatment periods, respectively. For methanogenic archaea, 64 OTUs were shared by both periods, while 33 and 1 unique OTUs were found in the control and treatment periods, respectively. For fungi, 642 OTUs were shared by both periods, while 748 and 107 unique OTUs were found in the control and treatment periods, respectively. Thus, the number of bacterial species increased with the addition of WGH extract, while the number of methanogenic archaeal and fungal species decreased.

**Figure 2:**
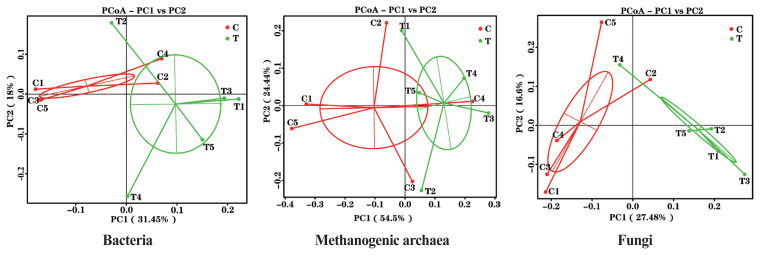
PCoA plots of ruminal bacteria, methanogenic archaea and fungi of sheep based on 97% similarity Weighted Unifrac distance matrices. The greater distances between samples indicate greater differences in microbial community composition. C means the control period, the animals were fed a basal diet; T means the treatment period, the animals were fed the basal diet supplemented with 0.5% hydroethanolic extract of walnut green husks; Numbers (1–5) represent the sample ID numbers; PCoA means principal co-ordinates analysis; PC1 means the first principal coordinate; PC2 means the second principal coordinate.

The effects of the hydroethanolic extract of WGH on the structure of the rumen microbial community are demonstrated in Figure 2. Principal co-ordinates analysis at OTU level based on weighted Unifrac distances showed that PC1 was 31.45%, 54.5%, and 27.48%, and PC2 was 18.00%, 24.44%, and 16.60% for bacteria, archaea, and fungi, respectively. The distance between the two periods showed that the addition of WGH extract had the greatest effect on the community structure of bacteria and fungi, and less on that of methanogenic archaea.

**Figure 3:**
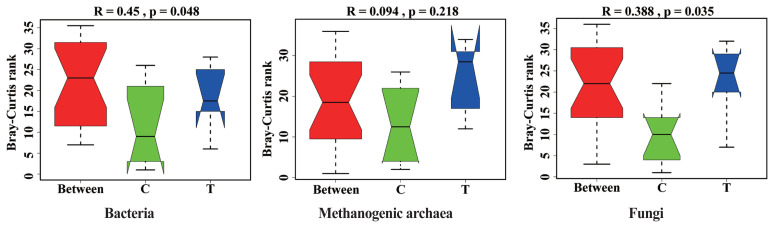
Analysis of similarity (Anosim) analysis of microbial community structure in sheep rumen. C means the control period, the animals were fed a basal diet; T means the treatment period, the animals were fed the basal diet supplemented with 0.5% hydroethanolic extract of walnut green husks; R means Pearson correlation coefficient; p means possibility. p<0.05 indicates a significant difference in microbial community structure between the two periods.

The effects of hydroethanolic extract of WGH on the microbial community structure are illustrated in Figure 3. Anosim analysis revealed a significant change in the community structure of bacteria and fungi after the addition of WGH extract (p<0.05). However, no significant changes were observed in the community structure of archaea (p>0.05).

### Relative abundance and biomarkers of rumen microorganisms

**Figure 4:**
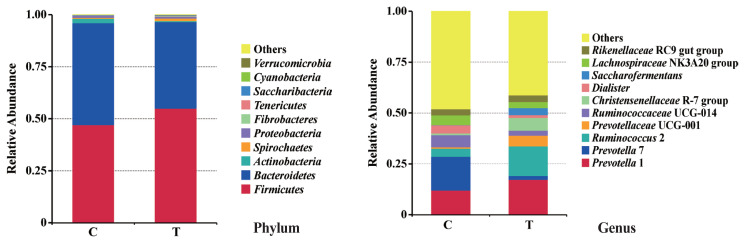
Effect of hydroethanolic extract of WGH on relative abundance of ruminal bacteria at phylum and genus level in sheep. C means the control period, the animals were fed a basal diet; T means the treatment period, the animals were fed the basal diet supplemented with 0.5% hydroethanolic extract of WGH. WGH, walnut green husks.

**Figure 5:**
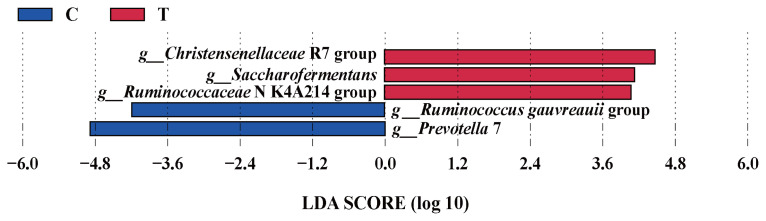
LEfSe analysis of biomarkers between groups of sheep ruminal bacteria. C means the control period, the animals were fed a basal diet; T means the treatment period, the animals were fed the basal diet supplemented with 0.5% hydroethanolic extract of WGH; LDA means linear discriminant analysis; *g*_ means genus. The positive values of LDA score (log10) indicate the significantly increased relative abundance of taxa (i.e. microbial biomarkers in the treatment period) compared to the control period, while the negative values of LDA score (log10) indicate the significantly decreased relative abundance of taxa (i.e. microbial biomarkers in the control period) compared to the treatment period. The threshold of 4 was set for LDA score (log10).

The effects of the hydroethanolic extract of WGH on the relative abundance of ruminal bacteria at the phylum and genus level are illustrated in Figure 4. At the phylum level, the *Firmicutes* and *Bacteroidetes* were the predominant bacteria. At the genus level, *Prevotella* and *Ruminococcus* were the predominant. The relative abundance of *Firmicutes*, *Prevotella* 1, *Prevotellaceae* UCG-001, and *Christensenellaceae* R-7 group increased slightly after the addition of WGH extract, while *Bacteroidetes*, *Prevotella* 7, and *Ruminococcaceae* UCG-014 showed a slight decrease in relative abundance. The search for the biomarkers between groups using LEfSe analysis revealed that the relative abundance of *Christensenellaceae* R7 group, *Saccharofermentans*, and *Ruminococcaceae* NK4A214 group increased significantly, while the relative abundance of *Ruminococcus gauvreauii* group, and *Prevotella* 7 decreased significantly after the addition of WGH extract (p<0.05) (Figure 5).

The effects of the hydroethanolic extract of WGH on the relative abundance of methanogenic archaea at the phylum and genus level are illustrated in Table 7. At the phylum level, the archaea in the rumen of sheep were dominated by *Euryarchaeota* with a relative abundance greater than 99.8%. At the genus level, *Methanobrevibacter* was the dominant archaea, with a relative abundance of more than 99.4%. The relative abundance of *Methanobrevibacter* was significantly increased by the addition of WGH extract (p<0.05). However, LEfSe analysis did not reveal any meaningful biomarkers between the two groups.

**Figure 6:**
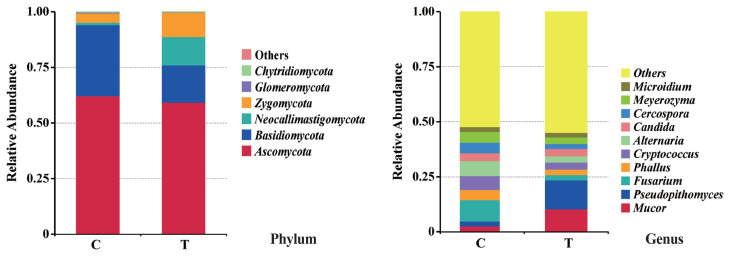
Effects of hydroethanolic extract of walnut green husk on relative abundance of ruminal fungi at phylum and genus level in sheep. C means the control period, the animals were fed a basal diet; T means the treatment period, the animals were fed the basal diet supplemented with 0.5% hydroethanolic extract of walnut green husks.

**Figure 7:**
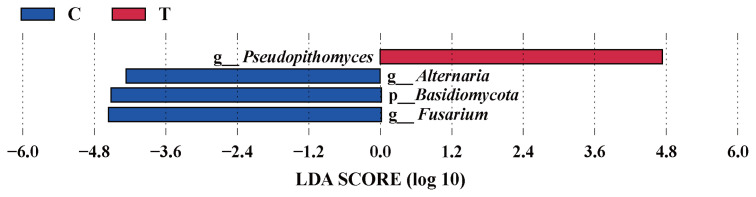
LEfSe analysis of biomarkers between groups of sheep ruminal fungi. C means the control period, during which the animals were fed a basal diet; T means the treatment period, during which the animals were fed the basal diet supplemented with 0.5% hydroethanolic extract of walnut green husks; LDA means linear discriminant analysis; p_ represents phylum; *g*_ represents genus. The positive values of LDA score (log10) indicate the significantly increased relative abundance of taxa (i.e. microbial biomarkers in the treatment period) compared to the control period, while the negative values of LDA score (log10) indicate the significantly decreased relative abundance of taxa (i.e. microbial biomarkers in the control period) compared to the treatment period. The threshold of 4 was set for LDA score (log10).

The effects of the hydroethanolic extract of walnut green husk on the relative abundance of rumen fungi at the phylum and genus level are illustrated in Figure 6. The dominant fungal phyla in sheep rumen were *Ascomycota*, *Basidiomycota*, *Neocallimastigomycota* and *Zygomycota*. During the control period, their abundance was 62.11%, 31.92%, 1.12%, and 4.20%, respectively. After the addition of WGH extract, their abundances were 59.21%, 16.72%, 12.72%, and 11.03%, respectively. Notably, there was a slight decrease in the relative abundance of *Basidiomycota*, accompanied by a slight increase in the relative abundances of *Neocallimastigomycota* and *Zygomycota*. At the genus level, the relative abundances of *Fusarium*, *Alternaria*, and *Cryptococcus* exceeded 5% in the control period, with 9.63%, 6.86%, and 6.29%, respectively. After the addition of WGH, only the relative abundance of *Mucor* and *Pseudopithomyces* exceeded 5%, with 10.36% and 13.18%, respectively. The relative abundance of *Fusarium*, *Alternaria*, and *Cryptococcus* decreased to 2.33%, 2.87%, and 3.19%, respectively. LEfSe analysis showed a significant increase in the relative abundance of *Pseudopithomyces* coupled with a significant decrease in the relative abundance of *Basidiomycota*, *Fusarium* and *Alternaria* after the addition of WGH extract (Figure 7).

## DISCUSSION

When SARA occurs in ruminants, it is usually associated with reduced dry matter intake, reduced production performance and increased animal culling rate, resulting in huge economic losses of the cattle and sheep industries. In the past, monensin was often used to control SARA in cattle and sheep, but due to the potential threats to food safety and human health, monensin has been banned as a feed additive in many countries, including the European Union and China. Plants and their extracts contain large amounts of phytochemicals such as saponins, alkaloids, phenolic compounds (e.g., tannins and flavonoids), terpenoids and essential oils with antioxidant and antibacterial effects, which play an active role in stabilizing rumen pH and preventing SARA [27]. Extracts of cinnamon (*Cinnamonum cassia*), grape seed (*Vitis vinifera*), orange peel (*Citrus sinensis*), pomegranate peel (*Punica granatum*), propolis and guava (*Psidium guajava*) significantly increased the pH of mixed rumen microorganisms in *in vitro* fermentation, cinnamon extract also reduced lactate and total VFA concentrations in rumen fluid and improved the ratio of acetate to propionate, and could be used as an alternative to monensin to effectively control rumen acidosis [2]. The addition of a mixture of mangosteen peel (*Garcinia mangostana* L.), rambutan peel (*Nephelium lappaceum* L.), banana flower powder (*Musa sapientum* L.) and cassava starch (*Manihot esculenta*, rich in condensed tannins and saponins) to the diet had a good buffering effect on the decrease in rumen fluid pH caused by high concentrate rations, and the rumen pH increased from 5.74 to 6.19 after 8 h of feeding, and nutrient digestibility and efficiency of microbial nitrogen supply were significantly improved, while ruminal methane production and protozoa were significantly reduced [28]. Overall, botanical extracts have been shown to have the ability to increase rumen fluid pH. However, their effect on VFA production is highly variable and largely depends on the nature and amount of active ingredients in the plant.

WGH is rich in gallic acid, protocatechuic acid, catechin, caffeic acid, ferulic acid, quercetin, kaempferol, hesperidin and other polyphenols as well as juglone, flavonoids, tetralone derivatives and naphthalene derivatives [3,29]. These compounds have good anticancer, antioxidant, antifungal and antibacterial properties. Chen et al [5] found that ethyl acetate extract of WGH decreased the enzyme activities of lactate dehydrogenase, hexokinase, and pyruvate kinase in rumen fluid, inhibited the proliferation of protozoa, decreased the production of acetate, propionate, butyrate, and lactate, thereby increasing the pH of rumen fluid and alleviating SARA induced by high-concentrate diet in sheep [5]. In the present study, the addition of hydroethanolic extract of WGH also elevated the pH of rumen fluid and showed good control of rumen SARA. However, its effect on VFA differed somewhat from that reported by Chen et al [5]. In this study, the addition of hydroethanolic extract of WGH showed significantly higher acetate, butyrate and isobutyrate, and significantly lower propionate and valerate than the control period, but no significant change in total VFA. The contradiction of the current study results with the result of Chen et al [5] could be related to the physicochemical properties of the extraction solvent because ethyl acetate extract contains more weak polar and non-polar compounds, while the hydroethanolic extract contains more polar small molecules. However, in the current study it has been observed that hydroethanolic extract of WGH contains a sufficient amount of rutin (Table 2) and the work of Oskoueian et al [30] reported an increase in acetate and slight decrease in propionate concentrations in the rumen by rutin. Similarly, Lowry and Kennedy [31] and McSweeney and Mackie [32] reported that rutin has the potential to increase the concentrations of acetate and butyrate in the rumen. Therefore, it could be assumed that higher acetate and lower propionate were due to the presence of rutin in the hydroethanolic extract of WGH and hence it could be speculated that hydroethanolic extract of WGH as a feed additive could effectively control the SARA in sheep.

It is generally believed that an increase in the concentration of acetate in the rumen results in an increase in methane production that could negatively influence the animal performance. Walnut husk also contain tannin, and tannin acts directly on rumen methanogens [33] and findings of lower methanogenic archaea in our study could be explained by tannin contents in WGH extract. It has been reported that tannin content decrease methane production by binding and/or penetrating the cell of methanogens thereby causing toxicity as has been suggested in previous study [34]. Tannins are also thought to directly inhibit methanogens, as well as indirectly limit methanogenesis through reduction of hydrogen availability [35]. There is also the possibility that a decrease in methanogens would increase the partial pressure of H_2_ in the rumen with negative effects on fibre degradation. However, *in vivo* studies with chemo-inhibitors have shown that decreases in CH_4_ and increases in gaseous H_2_ emissions has no negative effects on animal production [36]. Thus, a reduction in methanogens with tannin does not necessarily imply negative effects on animal performance.

It was found that a diet rich in walnuts significantly increased the abundance of intestinal *Ruminococcaceae* and *Bifidobacteria* and significantly decreased the abundance of *Clostridium* sp. cluster XIVa species (mainly *Blautia* and *Anaerostipes*) in healthy individuals [37]. The abundance of probiotic bacteria such as *Lactobacillus* spp., *Ruminococcus* spp. and *Roseburia* spp. was significantly higher in rats consuming a walnut-containing diet for 10 weeks [38]. WGH ethanol extract reduced the ratio of *Firmicutes*/*Bacteroidetes* and the relative abundance of potentially pathogen such as *Lachnospiraceae* and increased the relative abundance of potentially beneficial bacteria *Muribaculaceae* in rats [39]. These studies suggested that the active compounds in walnuts have beneficial effects in regulating the gut microflora. The effect of WGH extract on the gut microflora of animals has not been reported. In this study, we found that the relative abundance of *Christensenellaceae* R7 group, *Saccharofermentans*, and *Ruminococcaceae* NK4A214 group was significantly increased by the addition of WGH extract. *Christensenellaceae* R-7 group is a member of the phylum *Firmicutes* and the family *Christensenellaceae*. *Christensenellaceae* is a relatively new family of bacteria involved in the positive regulation of the gut environment and is associated with host immune regulation and healthy homeostasis *in vivo*, and thus are potentially beneficial bacteria [40]. In ruminants, the *Christensenellaceae* R-7 group promoted rumen development and increased digestion and absorption of nutrients [41]. *Saccharofermentans*, a member of the *Firmicutes*, can degrade fiber and utilize glucose to produce acetate, succinate, and lactate [42]. In the present study, one of the reasons for the increase in acetate may be the result of an increase in the relative abundance of *Saccharofermentans*. *Ruminococcaceae* belongs to the phylum *Firmicutes*, which was considered as fibrolytic bacteria that ferment the complex component of the plant cell wall to produce VFAs. The increase in *Ruminococcaceae* NK4A214 group indicated the beneficial effects of WGH extract on cellulolytic bacteria. Based on the available literature and the results of this experiment, the effects of WGH extract on the gut microbiome are mainly involved in fiber degradation, production of short-chain fatty acids, and maintenance of gut health.

In the present study, the relative abundance of *Ruminococcus* gauvreauii group and *Prevotella* 7 decreased significantly with the addition of WGH extract. *Ruminococcus gauvreauii* group, a member of *Firmicutes*, which was first isolated from human feces, and its role in the rumen of ruminants is very poorly understood. Some strains of the *Ruminococcus gauvreauii* group degrade mucin to produce propionate, which provides energy to the host and promotes their own colonization. Research has shown that quercetin inhibits the growth of *Ruminococcus gauvreauii*, *Bacteroides galacturonicus*, and *Lactobacillus* sp. [43]. Quercetin is one of the major phenolic compounds found in walnut husk. The decrease in the *Ruminococcus gauvreauii* group in this study may be related to the richness of WGH in phenolic compounds such as quercetin. *Prevotella* is a human conditional pathogen that can cause problems such as inflammation of tissues and organs. *Prevotella* helps the host digest carbohydrates such as arabinoxylan and oligofructose to produce propionate [44]. It has been reported that flavonoids supplementation reduces the abundances of *Prevotella* and concentration of propionate in the rumen and these findings are in line with our results [45]. It has also been reported that the abundance of *Prevotella* is negatively associated with the ruminal pH [46], hence in this study, increased ruminal pH and higher concentration of total VFA by supplementation of hydroethanolic extract of WGH may have caused a decrease in the relative abundance of *Prevotella*.

When the pH of rumen fluid decreased below 5.6, there was a significant death of protozoa and a decrease in the number of fibrolytic bacteria [47]. In the present study, the addition of WGH extract resulted in a significant increase in the number of protozoa, as well as the activities of FPase and cellobiase, and a tendency to increase xylanase activity, which may be because the addition of WGH extract increased the ruminal pH and improved the microbial living environment, protozoa, gram-negative bacteria, and fibrolytic bacteria were able to proliferate in the rumen.

*Ascomycota*, *Basidiomycota*, and *Neocallimastigomycota* are the major fungi in the rumen of yaks, cows, and other ruminants. Thus, these three may be the core fungi of bovidae species that play an important role in rumen digestion [48]. *Basidiomycota* produce β-glucanase and are the major lignocellulolytic fungi in the rumen, which can improve feed digestibility even at low abundance [49]. In the present study, the major genera of rumen fungi were *Mucor*, *Pseudopithomyces*, *Fusarium*, *Cryptococcus*, and *Alternaria*. The genera *Pseudopithomyces*, *Fusarium*, and *Alternaria* are all members of the phylum *Ascomycota*. *Mucor* is a member of the phylum *Zygomycota*, and *Cryptococcus* is a member of the phylum *Basidiomycota*. There are no reports about the role of these genera in rumen digestion and metabolism so far.

## CONCLUSION

The addition of hydroethanolic extract of WGH to a high-concentrate diet improved the ruminal microbial fermentation by increasing the activity of some carbohydrate digestive enzymes and the acetate to propionate ratio and altered the structure of ruminal bacterial and fungal communities. This may indicate that hydroethanolic extract of WGH can be a beneficial additive to alleviate SARA of sheep.

## Figures and Tables

**Table 1:** Composition and nutrient levels of basal diet (dry matter basis, %)

Items	Content
Ingredients	
Corn	40.79
Cottonseed meal	12.61
Soybean meal	4.18
Wheat bran	3.35
Urea	1.13
Limestone	1.06
NaCl	0.57
CaHPO_4_	1.26
Premix^1)^	0.05
Corn stover	35
Total	100
Nutrient levels^2)^	
Dry matter	94.09
Organic matter	92.26
Gross energy (MJ/kg)	17.17
Crude protein	17.91
Calcium	0.91
Phosphorus	0.3
Neutral detergent fiber	50.55
Acid detergent fiber	15.21

1) The premix provided the following per kg of diets: Vit A 1,350 IU, Vit D 270 IU, Vit E 45 IU, Fe 16 mg, Cu 8 mg, Zn 5 mg, Mg 10 mg, I 8.5 mg, Co 0.10 mg, Se 0.20 mg.

2) Nutrient levels are measured values.

**Table 2:** Active ingredients and free radical scavenging rate of hydroethanolic extract of walnut green husks

Active ingredients and free radical scavenging rate	Contents
Polysaccharides (%)	27.84
Flavonoids (mg rutin equivalents/g dry matter)	29.93
Total phenols (mg gallic acid equivalents/g dry matter)	60.07
DPPH free radical scavenging rate (%)	69.16

DPPH, 2,2-diphenyl-1-picrylhydrazyl.

**Table 3:** Primers used for polymerase chain reaction

Microorganisms	Regions	Primers (5′-3′)	References
Bacteria	V3–V4	341F (5′-CCTAYGGGRBGCASCAG-3′)	[20]
		806R (5′-GGACTACNNGGGTATCTAAT-3′)	
Methanogenic archaea	V8	1106F (5′-TTWAGTCAGGCAACGAGC-3′)	[21]
		1378R (5′-TGTGCAAGGAGCAGGGAC-3′)	
Fungi	ITS1	ITS5-1737F (5′-GGAAGTAAAAGTCGTAACAAGG-3′)	[22]
		ITS2-2043R (5′-GCTGCGTTCTTCATCGATGC-3′)	

**Table 4:** Effects of hydroethanolic extract of walnut green husks on voluntary intake and ruminal fermentation of sheep

Index	Control period	Treatment period	SEM	p-value
Feed intake (kg/d)	1.92	2.04	0.065	0.276
pH	5.44	5.68	0.042	0.005
Total VFA (mmol/L)	123.23	131.05	3.841	0.111
Acetate (mmol/L)	60.33	77.56	3.309	0.006
Propionate (mmol/L)	43.92	31.64	0.628	<0.001
Butyrate (mmol/L)	14.85	18.65	1.029	0.021
Valerate (mmol/L)	2.84	1.65	0.221	0.006
Isobutyrate (mmol/L)	0.42	0.54	0.043	0.045
Isovalerate (mmol/L)	0.87	1.01	0.144	0.084
Acetate/propionate	1.50	2.64	0.157	0.002
NH_3_-N (mg/L)	27.44	33.68	2.414	0.061
MCP (mg/mL)	8.46	9.20	1.945	0.726
Protozoa (log_10_[counts], mL)	6.54	6.95	0.023	<0.001

SEM, standard error of the means; VFA, volatile fatty acid; MCP, microbial crude protein.

**Table 5:** Effects of hydroethanolic extract of walnut green husks on digestive enzyme activity in rumen fluid

Enzymes	Control period	Treatment period	SEM	p-value
CMCase (IU/mL)	5.26	6.10	0.633	0.257
FPase (IU/mL)	0.30	0.52	0.015	<0.001
Cellobiase (IU/mL)	1.78	2.96	0.090	<0.001
Xylanase (IU/mL)	4.93	12.20	2.993	0.072
β-Glucanase (IU/mL)	3.66	7.97	2.335	0.141
Amylase (IU/mL)	6.40	6.86	0.890	0.634

SEM, standard error of the means; CMCase, carboxymethylcellulase; FPase, filter paper cellulase.

**Table 6:** Effects of hydroethanolic extract of walnut green husks on diversity indices of ruminal microbiome

Index	Control period	Treatment period	SEM	p-value
Bacteria				
Number of OTUs	509.40	523.20	60.926	0.832
Shannon	5.63	5.85	0.761	0.789
Simpson	0.93	0.95	0.029	0.562
Chao1	548.17	572.82	48.739	0.640
ACE	554.37	567.24	49.810	0.809
PD whole tree	40.72	43.02	3.691	0.566
Methanogenic archaea				
Number of OTUs	67.80	47.80	7.036	0.047
Shannon	2.88	2.39	0.120	0.015
Simpson	0.76	0.68	0.037	0.083
Chao1	71.58	51.13	6.518	0.035
ACE	71.83	51.13	6.593	0.039
PD whole tree	3.35	2.02	0.480	0.051
Fungi				
Number of OTUs	605.00	417.00	59.955	0.035
Shannon	6.53	6.04	0.253	0.123
Simpson	0.97	0.96	0.011	0.439
Chao1	634.93	446.45	52.336	0.023
ACE	627.95	448.71	53.753	0.029
PD whole tree	160.04	99.28	14.706	0.014

SEM, standard error of the means; OTUs, operational taxonomic units; ACE, abundance-based coverage estimator; PD, phylogenetic diversity.

**Table 7:** Effects of hydroethanolic extract of walnut green husks on relative abundance of methanogenic archaea at phylum and genus level in sheep

Taxa (%)	Control period	Treatment period	SEM	p-value
Phylum				
*Euryarchaeota*	99.87	99.98	0.048	0.068
*Thaumarchaeota*	0.093	0.013	0.031	0.068
Others	0.040	0.003	0.016	0.138
Genus				
*Methanobrevibacter*	99.43	99.82	0.157	0.043
*Methanosphaera*	0.178	0.132	0.060	0.345
*Methanocella*	0.041	0.003	0.016	0.144
*Methanobacterium*	0.032	0.003	0.012	0.144
*Methanoregula*	0.026	0.003	0.012	0.109
*Methanosaeta*	0.026	0.003	0.011	0.104
*Methanosarcina*	0.028	0.008	0.009	0.225
*Candidatus_Methanoperedens*	0.023	0.002	0.009	0.080
*Methanimicrococcus*	0.011	0.002	0.007	0.273
*Candidatus_Nitrosotalea*	0.013	0	0.005	0.109
Others	0.187	0.021	0.054	0.043

SEM, standard error of the means.
